# Drug-resistant bacterial infections in end-stage liver disease: immune imbalance and intervention advances

**DOI:** 10.3389/fimmu.2026.1888293

**Published:** 2026-08-17

**Authors:** Jing Liu, Jun Wu, Xin Zheng

**Affiliations:** Department of Infectious Diseases, Union Hospital, Tongji Medical College, Huazhong University of Science and Technology, Wuhan, China

**Keywords:** drug-resistant bacterial infection, end-stage liver disease, gut microbiota, immune checkpoint, immune dysfunction, immunometabolism

## Abstract

Patients with end-stage liver disease (ESLD) face a markedly elevated risk of infections caused by multidrug-resistant (MDR) and extensively drug-resistant (XDR) bacteria. This vulnerability worsens prognosis and severely limits therapeutic options. The underlying mechanisms extend beyond hepatic synthetic and detoxification failure to encompass a complex and multifaceted state of immune dysfunction. In this review, we synthesize current evidence on how ESLD-associated immune defects drive susceptibility to drug-resistant infections. We examine key abnormalities across multiple interconnected domains: depletion and dysfunction of Kupffer cells, complement deficiency, T-cell exhaustion with regulatory T-cell expansion, disruption of the gut-liver axis, immunometabolic reprogramming driven by hyperammonemia and lactate accumulation, and upregulation of immune checkpoint molecules such as PD-1/PD-L1. These pathways collectively promote colonization, persistence, and therapeutic refractoriness of MDR pathogens. We also evaluate emerging therapeutic strategies targeting these immune defects, including checkpoint inhibitors, cytokine and cellular therapies, microbiome modulation, and metabolic interventions, while acknowledging the challenges that limit their clinical translation. By proposing an integrated framework that links distinct immune defects to MDR infection pathogenesis, we aim to guide the development of biomarker-driven, personalized immunomodulatory approaches that complement antimicrobial therapy and ultimately improve outcomes in this high-risk population.

## 1 Introduction

**Figure 1:**
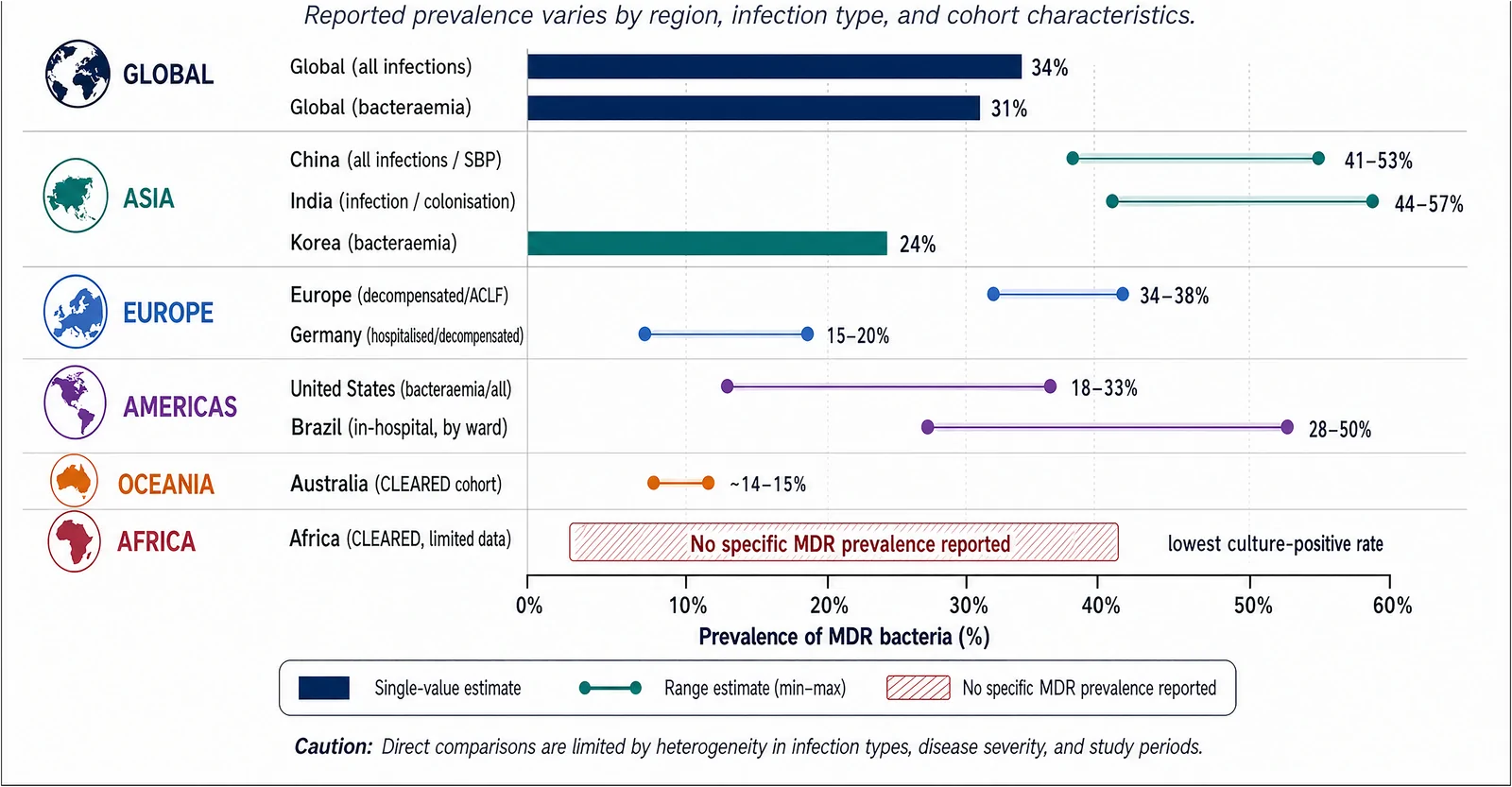
Country-level prevalence of multidrug-resistant (MDR) bacteria in patients with ESLD. The map summarizes reported MDR prevalence among cirrhotic or ESLD patients from large−scale epidemiological studies, meta−analyses, and multicenter cohorts published between 2019 and 2026. Global estimates are 34% for all infections and 31% for bacteraemia, but regional rates differ substantially across study settings and patient populations. China reports 41–53%, India 44–57%, Korea 24%, Europe 34–38%, Germany 15–20%, the United States 18–33%, Brazil 28–50% depending on hospital ward, and Australia approximately 14–15%. For Africa, no specific MDR rate has been reported, although the CLEARED study noted the lowest culture−positive rate in this region, suggesting possible under−detection rather than true absence of resistance. These comparisons should be interpreted with caution, given heterogeneity in study design, infection type, disease severity, and time period.

End-stage liver disease (ESLD), encompassing decompensated cirrhosis and acute-on-chronic liver failure (ACLF), represents a formidable global health challenge, characterized by high morbidity and mortality rates. Patients with ESLD are exceptionally vulnerable to infections, particularly those caused by multidrug-resistant (MDR) and extensively drug-resistant (XDR) organisms. Globally, MDR bacteria account for 34% of all infections and 31% of bacteraemia cases in patients with cirrhosis (1, 2). Regional estimates vary markedly: China 41–53% (3, 4), India 44–57% (5, 6), Korea 24% (7), Germany 15–20% (8, 9) and other European countries 34–38% (10), the USA 18–33% (1, 11), Brazil 28–50% (12), and Africa with under−detection (13) (**Figure 1**). This susceptibility stems from a confluence of clinical and pathophysiological factors, including frequent hospitalizations, exposure to invasive procedures, prolonged or repeated courses of broad-spectrum antibiotics, and, most critically, a profound and complex state of systemic immune dysfunction. Infections in this population are not merely incidental complications; they are pivotal events that often precipitate clinical deterioration, leading to sepsis, accelerated hepatic decompensation, and a dramatic increase in short-term mortality. The management of these infections is further complicated by the limited therapeutic arsenal against MDR/XDR pathogens and the altered pharmacokinetics of antimicrobial agents in liver failure, creating a perfect storm that underscores the urgent need for a deeper mechanistic understanding.

Previously, the heightened infection risk in cirrhosis and liver failure was largely attributed to quantitative defects in the innate immune system, such as neutropenia due to hypersplenism and reduced synthesis of complement proteins by the failing liver. While these factors remain relevant, contemporary research has unveiled a far more intricate and paradoxical immunopathology. ESLD is now recognized as a state of concurrent “immune paralysis” and “immune activation” a dysregulated equilibrium affecting both innate and adaptive immunity. On one hand, there is a functional impairment or exhaustion of immune effector cells. This phenomenon is often termed “immunoparalysis”. This includes impaired neutrophil chemotaxis and phagocytosis, reduced oxidative burst capacity, monocyte dysfunction with diminished human leukocyte antigen (HLA)-DR expression and cytokine production in response to pathogens, and T-cell exhaustion marked by increased expression of inhibitory receptors like programmed cell death 1 (PD-1). On the other hand, a baseline state of systemic inflammation and immune activation persists, driven by persistent exposure to gut-derived pathogen-associated molecular patterns (PAMPs) and damage-associated molecular patterns (DAMPs) due to increased intestinal permeability and impaired hepatic clearance.

This dual state of immunosuppression and chronic inflammation creates a uniquely permissive environment for bacterial infections and profoundly influences the dynamics of pathogen selection. The impaired clearance capacity allows initial colonization and infection to establish more easily. Simultaneously, the inflammatory milieu, along with the selective pressure exerted by antibiotic use, may inadvertently favor the emergence and proliferation of resistant strains. For instance, certain inflammatory cytokines can modulate bacterial gene expression, potentially upregulating efflux pumps or biofilm formation. Moreover, the dysregulated immune response fails to mount an effective, coordinated attack, allowing infections to become persistent and recurrent, thereby increasing cumulative antibiotic exposure and further driving resistance. In this context, antibiotic stewardship principles specifically tailored to cirrhosis are of critical importance. Current guidelines recommend risk−based empiric antibiotic selection, de−escalation based on culture results, and limitation of treatment duration in patients with cirrhosis (10). The choice of empiric regimens must account for local resistance patterns, prior antibiotic exposure, healthcare−associated infection status, and illness severity, as inappropriate initial therapy has been consistently associated with increased mortality (14). Thus, the immune dysfunction in ESLD is not a passive bystander but an active participant in shaping the microbial landscape toward more resistant and virulent profiles.

Given this complex interplay, moving beyond the traditional paradigm of mere immune deficiency is crucial. A precise dissection of the specific immune pathways that are disrupted in ESLD and that directly or indirectly facilitate MDR/XDR infections is essential. This involves exploring how components such as Kupffer cells (KCs), mucosal−associated invariant T cells, the gut−liver axis, and the balance between pro−inflammatory and anti−inflammatory signaling contribute to both susceptibility and poor outcomes.

Despite growing recognition of immune dysfunction as a key driver of infection susceptibility in ESLD, several critical knowledge gaps remain. The precise mechanisms by which specific immune defects facilitate the colonization and persistence of MDR pathogens are not fully elucidated. The interplay between these immune domains also remains poorly understood in the ESLD context, particularly regarding how innate immune failure aggravates adaptive dysfunction or how metabolic disturbances modulate checkpoint expression. Another unresolved question concerns which immune alterations are most amenable to therapeutic intervention and whether such interventions can improve infection−related outcomes beyond antibiotic therapy alone.

This review addresses these gaps by systematically synthesizing current evidence across the innate and adaptive immune systems, the gut−liver axis, immunometabolism, and immune checkpoints. We propose an integrated framework that links specific immune defects to MDR infection pathogenesis. Unraveling these mechanisms holds the key to identifying novel diagnostic biomarkers that can stratify infection risk or predict outcomes more accurately than current clinical scores. More importantly, it opens the door to innovative therapeutic strategies aimed at rebalancing the host response rather than solely targeting the pathogen, which could serve as adjunctive therapies to antibiotics in the era of diminishing antimicrobial efficacy. The immune pathways dissected here are not merely of theoretical interest; they represent actionable targets for diagnostic and therapeutic intervention. By identifying which immune defects are most closely tied to MDR infection risk, we aim to inform the development of biomarker-based strategies for risk stratification and to guide the rational design of immunomodulatory adjunctive therapies. Through this comprehensive analysis, we hope to identify promising immunomodulatory strategies and highlight priority areas for future translational research.

## 2 Defects and dysfunction of the innate immune system

In ESLD, the innate immune system exhibits profound and multifaceted dysfunction, critically compromising the host’s first line of defense against infections. The immune pathways affected in ESLD vary depending on the underlying cause of liver disease. In alcohol−associated liver disease, chronic ethanol exposure impairs intestinal barrier function, allowing increased lipopolysaccharide translocation and persistent Toll−like receptor 4 (TLR4) activation on Kupffer cells and hepatic stellate cells. This sustained activation eventually leads to TLR4 tolerance and immune exhaustion (15). In metabolic dysfunction−associated steatotic liver disease, TLR4 is primarily engaged by free fatty acids and metabolic endotoxemia, with additional input from TLR2 and TLR9 (16). In chronic HBV and HCV infections, viral proteins directly interfere with TLR signaling: HBV suppresses TLR3 and TLR4 expression, whereas HCV impairs TLR7 and TLR9 function, resulting in defective interferon responses (17, 18). Despite these distinct pathways, the final common outcome across all etiologies is a state of innate immune paralysis marked by impaired TLR signaling, defective pathogen recognition, and blunted antimicrobial effector function. This paralysis substantially increases susceptibility to MDR infections as liver disease progresses to ESLD.

A central component of this dysfunction is the impairment of myeloid cells, particularly KCs, the liver-resident macrophages. KCs are strategically positioned within sinusoids to form an intravascular “firewall” capturing and clearing blood-borne pathogens (19). However, in ESLD and acute liver injury, this firewall is severely compromised. KCs can be depleted through various cell death pathways, including apoptosis, pyroptosis, and a unique form of cell death known as PANoptosis, which is driven by factors like elevated heme during sepsis and involves mitochondrial damage (20, 21). This depletion creates a transient niche disruption, opening a window of susceptibility to systemic infections. Furthermore, even when present, KC function is severely suppressed. Studies show that during liver injury, KCs upregulate immune checkpoint molecules like PD-1, which leads to an immunosuppressive profile and a marked reduction in their antimicrobial responses and bacterial clearance capacity (22). Consequently, KC dysfunction turns the liver sinusoid into a sanctuary where resistant organisms proliferate and disseminate. The functional impairment extends to pathogen recognition. The solute carrier protein Slc2a6 in KCs has been shown to attenuate the capture of *Streptococcus pneumoniae* by downregulating crucial natural antibodies, thereby compromising early host defense (23). Additionally, the formation of pathological complexes, such as those between C-reactive protein and very low-density lipoproteins (CRP-VLDL), can competitively inhibit the phagocytosis of bacteria by activated myeloid cells, further delaying bacterial clearance (24). Beyond phagocytes, the complement system, whose proteins are predominantly synthesized in the liver, is severely depleted in ESLD. This limits the activation of all complement pathways, crippling key effector functions like opsonization (to promote phagocytosis) and membrane attack complex formation (for direct bacterial lysis), mechanisms especially critical for controlling Gram-negative MDR strains.

Beyond the intrinsic defects of KC and complement, the inflammatory environment in ESLD can further amplify tissue injury through intercellular crosstalk and inflammatory signaling cascades. In metabolic dysfunction−associated steatohepatitis, macrophage-neutrophil crosstalk via the Selplg-Sell-YAP axis has been shown to drive NETosis and fibrosis, suggesting that similar interactions may contribute to the progression of liver injury in ESLD (25). Likewise, inflammatory pathways such as JAK1/STAT3 have been implicated in acute liver injury; in a D-GalN/LPS-induced model, inhibition of this pathway attenuated liver damage, highlighting the potential of targeting such signaling cascades to mitigate hepatic injury (26). These additional mechanisms, while not directly affecting KC-mediated bacterial clearance itself, reinforce the overall tissue-damaging milieu that compounds the susceptibility to infection and impedes recovery. This collective innate immune paralysis, characterized by KC depletion/dysfunction and complement deficiency, creates a permissive environment for the onset and spread of MDR bacterial infections in ESLD patients.

The defects in innate immunity described above do not operate in isolation; they directly shape and exacerbate the dysfunction of the adaptive immune system. Impaired Kupffer cell-mediated bacterial clearance and complement deficiency lead to persistent antigenic stimulation and chronic inflammation, which in turn drive T-cell exhaustion and dysregulated humoral responses. Thus, innate immune failure sets the stage for the adaptive immune paralysis discussed below.

## 3 Suppression and exhaustion of the adaptive immune system

Building upon the innate immune defects outlined in Section 1, the adaptive immune system in ESLD is also characterized by profound suppression, exhaustion, and imbalance, rendering the host incapable of mounting effective pathogen-specific responses. This dysfunction is rooted in both quantitative and qualitative defects in T and B lymphocytes. A hallmark is the dysregulation of CD4+ T helper (Th) cell subsets, with a shift away from protective effector responses towards an immunosuppressive state. The balance between pro-inflammatory Th17 cells and anti-inflammatory regulatory T cells (Tregs) is particularly crucial for hepatic immune homeostasis, and its disruption is implicated in various liver diseases (27). In ESLD and associated conditions like sepsis, there is often an expansion of Tregs and a concomitant impairment of effector Th1 and Th17 cell functions.

This Treg-dominant environment has direct consequences for the control of MDR pathogens. Tregs suppress effector T-cell proliferation and cytokine production through multiple mechanisms, including the secretion of IL-10 and TGF-β, cell-contact-dependent inhibition via CTLA-4, and metabolic disruption through CD25-mediated IL-2 deprivation. By dampening Th17-mediated mucosal immunity and Th1-dependent intracellular bacterial clearance, the expanded Treg population creates a permissive niche for MDR organisms that rely on the host’s weakened adaptive response to establish chronic colonization and evade clearance (28, 29).

Immunometabolic factors dynamically regulate this Treg/Th17 equilibrium. Previous study have demonstrated that following liver transplantation, distinct immune cells undergo dynamic adaptive changes in energy metabolism that directly determine the outcomes of rejection, ischemia-reperfusion injury, and immune tolerance (30). In the ESLD liver microenvironment, alterations in lipid metabolism, glucose metabolism, and mitochondrial function can skew CD4+ T-cell differentiation toward Tregs while impairing Th17 effector functions, further reinforcing the immunosuppressive state that favors MDR pathogen persistence.

T cells in the cirrhotic and septic milieu exhibit an exhausted phenotype, with upregulated expression of inhibitory receptors like PD-1, which dampens their proliferative capacity and cytokine production (22). Exhausted T cells cannot effectively orchestrate antibacterial responses. This deficit becomes critical when antibiotics fail against MDR strains. The humoral arm of adaptive immunity is equally compromised. While ESLD patients often exhibit hyperglobulinemia, this reflects nonspecific polyclonal stimulation rather than effective humoral immunity. The liver’s synthetic failure and splenic hyperactivity lead to impaired B cell differentiation into plasma cells, resulting in the production of antibodies with low affinity and poor opsonizing capability. Consequently, even when pathogens are opsonized, the quality of the antibody response is insufficient for effective neutralization and clearance. Vaccination studies highlight the critical role of high-quality, functional antibodies in mediating protection. Effective vaccines against invasive bacteria work by inducing antibodies that orchestrate pathogen clearance in the liver, either through direct capture by KCs via Fcγ receptors or by activating complement for uptake via complement receptors (31). The failure to generate such robust, high-titer functional antibodies in ESLD patients leaves them vulnerable to MDR pathogens that would otherwise be controlled by vaccine-elicited immunity.

**Figure 2:**
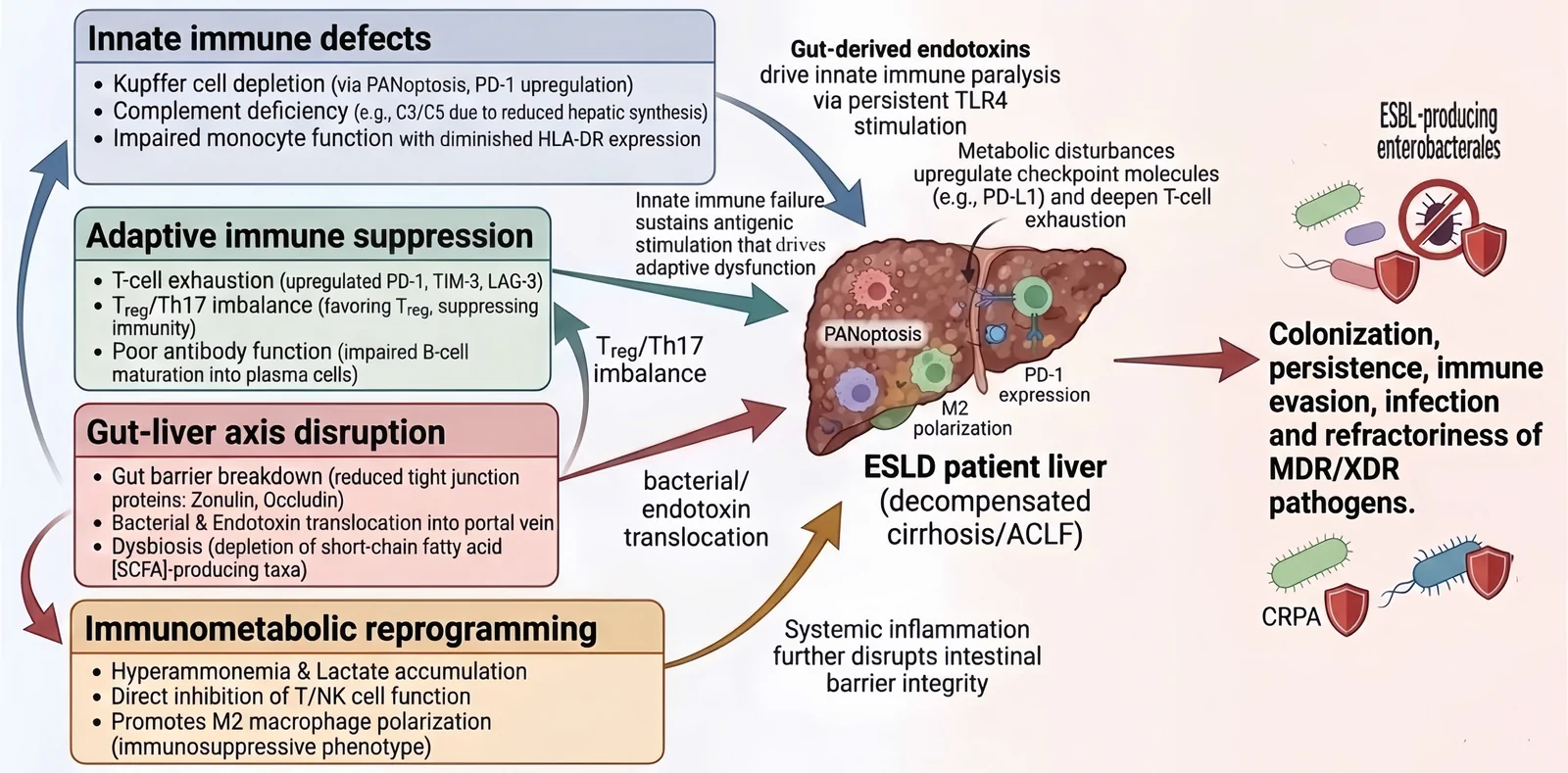
Core mechanisms of immune dysfunction and susceptibility to drug−resistant bacterial infections in end−stage liver disease (ESLD). The diagram depicts four interconnected components of immune dysfunction that collectively drive susceptibility to multidrug-resistant (MDR) and extensively drug-resistant (XDR) pathogens in ESLD. The central liver represents the ESLD milieu, in which chronic inflammation and immune paralysis coexist. The first component, innate immune defects, includes Kupffer cell depletion via PANoptosis and PD-1 upregulation, complement deficiency due to reduced hepatic synthesis, and impaired monocyte function with diminished HLA-DR expression. These defects compromise the liver’s intravascular firewall and impair early pathogen recognition and clearance. The second component, adaptive immune suppression, features T-cell exhaustion marked by upregulated inhibitory receptors such as PD-1 and TIM-3, an imbalance between regulatory T cells and Th17 cells that favors immunosuppression over protective immunity, and poor antibody function resulting from impaired B-cell differentiation into plasma cells. The third component, gut–liver axis disruption, encompasses gut barrier breakdown with reduced tight junction protein expression, bacterial and endotoxin translocation into the portal circulation, and dysbiosis characterized by depletion of short-chain fatty acid-producing taxa. The fourth component, immunometabolic reprogramming, involves hyperammonemia and lactate accumulation, which together inhibit T-cell and natural killer cell function and promote M2 macrophage polarization toward an immunosuppressive phenotype. These four components are not independent. Arrows indicate mutual reinforcement among them: endotoxemia from gut barrier failure exacerbates innate immune paralysis, metabolic disturbances upregulate checkpoint molecules and deepen T-cell exhaustion, and persistent inflammation further disrupts intestinal integrity. Their convergence leads to colonization, infection, and poor clearance of MDR/XDR pathogens, ultimately contributing to the refractory nature of drug-resistant infections in ESLD patients.

Thus, the adaptive immune landscape in ESLD is one of functional paralysis, characterized by T cell exhaustion, a skewed Treg/Th17 balance that favors suppression over defense, and a dysfunctional B cell response that produces abundant but ineffective antibodies, collectively crippling the body’s ability to develop immunological memory and mount specific defenses against resistant pathogens (**Figure 2**).

## 4 Gut barrier disruption and bacterial translocation

In ESLD, the disruption of the gut barrier and subsequent bacterial translocation constitute a fundamental pathophysiological process that drives disease progression and predisposes to infections, including those caused by multidrug-resistant organisms (MDROs). Portal hypertension, a hallmark of advanced cirrhosis, leads to intestinal mucosal congestion and edema, which directly compromises the integrity of the intestinal epithelial barrier (32). This structural damage is associated with a reduction in the expression of key tight junction proteins, such as occludin and ZO-1 (33). The resultant increase in intestinal permeability facilitates the translocation of live bacteria and their products, such as lipopolysaccharide (LPS), from the gut lumen into the mesenteric lymph nodes and portal circulation (34). This process of bacterial translocation is a central mechanism linking gut dysbiosis to systemic inflammation and hepatic complications in cirrhosis (35). Dysbiosis, characterized by an overgrowth of potentially pathogenic bacteria and a decrease in beneficial, short-chain fatty acid (SCFA)-producing taxa like *Firmicutes* and *Clostridium*, is prevalent in ESLD and further erodes barrier function (36). Beyond alterations in SCFA-producing taxa, recent work has identified that a decrease in the commensal bacterium Flavonifractor plautii and its metabolite phytosphingosine is associated with metabolic disorders, suggesting that the depletion of specific microbial species and their bioactive products may contribute to the metabolic disturbances observed in ESLD (37). The translocation of microbial components, including endotoxin, persistently activates hepatic immune cells, particularly KCs, via TLR4 signaling, creating a state of chronic, low-grade systemic inflammation that underpins cirrhosis-associated immune dysfunction (38). This same pathophysiological cascade, driven by gut barrier disruption and bacterial translocation, also contributes to the development of hepatic encephalopathy (HE), a severe neuropsychiatric complication of cirrhosis. HE encompasses a range of neurological and psychiatric abnormalities resulting from hepatic insufficiency or portal-systemic shunting, with clinical manifestations varying from subtle cognitive impairment to deep coma (39). The gut–liver–brain axis is central to its pathogenesis. Dysbiosis and increased intestinal permeability allow bacterial products, particularly ammonia and endotoxins, to enter the systemic circulation (40). This triggers systemic inflammation and neuroinflammation, which disrupt blood–brain barrier integrity and impair cerebral metabolic function. These observations indicate that gut barrier failure in ESLD contributes not only to systemic infections but also to neurocognitive impairment, reinforcing the need to preserve intestinal barrier integrity in this population.

Critically, this environment, combined with the frequent and often broad-spectrum antibiotic use required in managing decompensated cirrhosis, creates a fertile ecological niche for MDROs. Antibiotic pressure and dysbiosis promote the colonization of the jejunum and colon with resistant pathogens such as vancomycin-resistant enterococci (VRE) and extended-spectrum β-lactamase-producing Enterobacterales (ESBL-E) (41). Once established, these MDROs can exploit the compromised intestinal barrier to translocate, leading to endogenous infections like spontaneous bacterial peritonitis (SBP) and bacteremia, which are major causes of morbidity and mortality in this population (42). The risk is particularly pronounced in patients awaiting or having undergone liver transplantation (LT), as they often have pre-existing dysbiosis and face additional perioperative insults, including immunosuppression and further antibiotic exposure, which can drastically alter gut microbiota composition and function (43). Recent studies have quantified the impact of pre-transplant MDR colonization on post-LT outcomes. In a cohort of 499 living-donor LT recipients, 32.6% were colonized with carbapenem-resistant Enterobacterales (CRE) pre-transplant, and preoperative CRE colonization was independently associated with postoperative Enterobacterales septicemia (44). Similarly, VRE colonization, present in approximately 20% of LT candidates, has been associated with increased risk of early acute kidney injury and clinically significant infections post-transplant (45). Targeted perioperative prophylaxis against MDR Gram-negative bacteria has been shown to significantly reduce infectious complications and infection-related mortality in colonized LT recipients (46), although the protective effect of novel agents against CRE infections may be limited to the first 15 days post-transplant (47). The post-LT period adds further complexity to the MDR challenge. Immunosuppression, surgical stress, and antibiotics create a dysbiotic gut microenvironment that promotes MDR pathogen expansion and translocation. Calcineurin inhibitors impair T-cell-mediated clearance and alter microbiota composition, favoring opportunistic pathogens. This state, combined with barrier disruption, allows translocating MDR bacteria to cause bacteremia, spontaneous bacterial peritonitis, and surgical site infections. Early post-transplant infections affect 30% to 60% of recipients, with MDR pathogens increasingly implicated (48). The gut microbiome evolves during the first year, with some patients recovering diversity while others remain in dysbiosis that sustains infection risk.

Thus, the triad of gut barrier breakdown, pathological bacterial translocation, and dysbiosis-driven MDRO colonization forms a vicious cycle that exacerbates liver disease, fuels systemic inflammation, and significantly elevates the risk of difficult-to-treat infections in patients with ESLD.

## 5 Regulation of hepatic immunity by gut-derived signals

The gut-liver axis exerts profound regulatory control over hepatic immunity in ESLD, primarily through the continuous delivery of microbial signals that shape a dysfunctional immune landscape conducive to infection and poor outcomes. A cornerstone of this dysregulation is the state of persistent, low-grade endotoxemia resulting from the translocation of LPS across the leaky intestinal barrier (34). The constant exposure of liver-resident macrophages (KCs) and hepatic stellate cells to LPS via TLR4 leads to a phenomenon of “TLR4 tolerance” or exhaustion, rendering these cells hyporesponsive to subsequent bacterial challenges (49). This immune paralysis, a key feature of cirrhosis−associated immune dysfunction (CAID), impairs the liver’s ability to mount an effective antimicrobial defense, a deficit that disproportionately benefits MDR organisms, as they rely heavily on host immune failure to survive when antibiotics are ineffective (50). Concurrently, the gut dysbiosis characteristic of ESLD results in a marked reduction in the production of beneficial microbial metabolites, particularly short-chain fatty acids (SCFAs) like butyrate by commensal bacteria such as *Firmicutes* and *Coprococcus (*36). SCFAs are crucial for maintaining intestinal epithelial integrity by promoting tight junction assembly and exerting potent anti-inflammatory effects both locally and systemically (51). Their depletion weakens the gut barrier, further facilitating bacterial translocation, and removes a critical regulatory signal for hepatic immune cells. Within the liver, SCFAs help modulate macrophage and T-cell function, promoting an anti-inflammatory, regulatory phenotype (52). SCFAs are not the only microbial metabolites with immunomodulatory properties. Phytosphingosine, a product of the commensal bacterium Flavonifractor plautii, has been shown to influence host metabolism, and its reduction has been linked to metabolic disorders (37). This observation highlights the broader relevance of the gut microbiome–metabolite axis in shaping host immunity and metabolism in ESLD. The loss of this regulatory influence, coupled with chronic endotoxin exposure, skews the hepatic immune milieu towards a pro-inflammatory, yet paradoxically immunosuppressed, state. This is evidenced by elevated levels of pro-inflammatory cytokines (e.g., IL-6, IL-17) and markers of systemic inflammation (e.g., C-reactive protein) in patients with decompensated cirrhosis and ACLF, which correlate with disease severity and infection risk (53). This environment favors MDR pathogen persistence and refractory infections, while post−transplant immunosuppressants like tacrolimus further disrupt gut microbiota and metabolites, raising MDR infection risk and compromising graft outcomes (54). Therefore, gut-derived signals (both the persistent incitement from endotoxin and the loss of protective modulation from SCFAs) orchestrate a hepatic immune environment characterized by concurrent inflammation and immune paresis. This imbalance not only accelerates liver disease progression but also critically undermines host defense, creating a permissive setting for the establishment and severity of infections, including those caused by antibiotic-resistant bacteria.

## 6 Energy metabolism disorders and immune cell function

ESLD is characterized by a profound systemic energy metabolism crisis, leading to a state of “immune cell starvation” where key immune effectors such as T cells and macrophages are deprived of the specific metabolic substrates required for their activation and effector functions. That selectively impairs the host’s ability to clear drug−resistant bacteria. This metabolic reprogramming is a central feature of liver diseases progressing to ESLD, including metabolic dysfunction-associated steatotic liver disease (MASLD) and alcohol-associated liver disease (ALD) (55, 56). In these conditions, the hepatic microenvironment undergoes significant metabolic alterations, such as deregulated amino acid and lipid metabolism and mitochondrial dysfunction, which collectively impair the immunometabolic responses of infiltrating and resident immune cells (55, 57). For T cells, the ESLD environment is particularly hostile. Factors like hyperammonemia and lactate accumulation, coupled with intrinsic mitochondrial abnormalities, severely disrupt critical metabolic pathways. Specifically, these disturbances interfere with glycolysis and glutaminolysis in T cells, leading to insufficient ATP production (58). This bioenergetic failure directly compromises the ability of T cells to undergo the necessary clonal expansion and execute cytotoxic or helper functions, a phenomenon observed in chronic viral hepatitis and other liver pathologies where T cell exhaustion is linked to mitochondrial impairment (59, 60). Concurrently, macrophages exhibit polarization abnormalities driven by altered metabolic substrate availability. Disturbances in arginine metabolism skew macrophages from the bactericidal M1 phenotype toward an immunosuppressive M2 phenotype (61, 62). This shift, evident in steatotic liver diseases, weakens the clearance of intracellular pathogens and facilitates immune tolerance (52, 63, 64). In the context of ESLD, M2−like predominance creates a permissive niche for MDR bacteria, such as ESBL−producing E. coli and VRE, which are otherwise poorly killed by residual immune defenses. Thus, the metabolic reprogramming of immune cells, deprived of essential fuels and skewed toward suppression, directly contributes to the persistence and recurrence of drug−resistant infections. This establishes a vicious cycle: liver injury drives metabolic dysfunction, which cripples immunity against MDR pathogens, allowing persistent infection that further exacerbates liver disease (56, 65).

## 7 Direct regulation of immune responses by metabolites

In ESLD, the accumulation of specific metabolic byproducts directly exerts immunosuppressive effects, shaping an immune−paralyzed state that selectively favors the persistence of drug−resistant bacteria. Hyperammonemia, a hallmark of advanced liver dysfunction, inhibits key metabolic enzymes and disrupts intracellular signaling cascades, leading to functional impairment of macrophages and T cells (58). Although direct evidence in ESLD patients is limited, studies from metabolic disorders and sepsis models corroborate these immunosuppressive effects (66, 67). This immune paresis is particularly detrimental for controlling MDR pathogens, which rely on host immune failure to survive when antibiotics are ineffective. Similarly, lactate accumulation creates a local immunosuppressive microenvironment akin to that found in solid tumors. In the context of ESLD, a “high lactate” state can develop due to factors such as tissue hypoxia, increased glycolytic flux in injured hepatocytes, and possibly impaired hepatic clearance (68, 69). This lactate−rich environment directly impairs natural killer (NK) cells, inhibiting proliferation, downregulating cytotoxic molecules, and disrupting mitochondrial bioenergetics (68, 70). Lactate also skews macrophage polarization toward an immunosuppressive M2 phenotype, reducing their bactericidal capacity against MDR strains (71, 72). Furthermore, lactate induces histone lactylation, an epigenetic modification that drives immunosuppressive gene expression programs, as documented in tumors and potentially in the inflamed liver (73, 74). The combined effects of ammonia and lactate, along with other dysregulated metabolites, establish a profoundly immunosuppressive milieu. This environment not only hampers the effector functions of NK cells and T cells but also actively supports the recruitment and function of regulatory immune cells, thereby creating a favorable niche for pathogen survival and replication in patients with ESLD (65, 75). Consequently, metabolic byproducts directly contribute to the persistence, recurrence, and poor antibiotic response of resistant infections.

## 8 Upregulation of co-inhibitory molecule expression

In the context of ESLD, the chronic inflammatory state and persistent antigenic stimulation characteristic of conditions like ACLF and hepatocellular carcinoma (HCC) drive a profound dysregulation of immune checkpoint molecules, leading to a state of T cell exhaustion and impaired antimicrobial immunity. A central mechanism involves the abnormal activation of the PD-1/PD-L1 pathway. Patients with ACLF show significantly increased PD−1 expression on CD8+ T cells, and PD−L1 engagement further suppresses their proliferation, activation, and cytokine production (76). This T cell dysfunction is particularly detrimental for controlling MDR pathogens, which rely on the host’s failed adaptive immunity to persist when antibiotics are ineffective. The source of PD−L1 is multifaceted: hepatocytes and biliary cells upregulate PD−L1 under inflammatory conditions, and circulating tumor cells in advanced HCC frequently express PD−L1, correlating with poor prognosis (77). The immunosuppressive network extends beyond PD−1. In cirrhotic patients awaiting liver transplantation, while baseline T cell checkpoint levels may not differ from healthy controls, there is significant expansion of LOX−1+ myeloid−derived suppressor cells (MDSCs) and immature neutrophils, especially during acute decompensation (78). MDSCs potently inhibit T cell function and create a microenvironment that favors colonization and persistence of MDR organisms. The collective upregulation of co−inhibitory receptors (PD−1, TIM−3, CTLA−4) on immune cells, coupled with MDSC expansion, establishes a powerful inhibitory network that dampens T cell effector functions. This network potently dampens T cell effector functions, leading to an exhausted state where T cells cannot effectively proliferate or produce cytokines, thereby crippling the host’s ability to mount an effective immune response against pathogens, including drug-resistant bacteria.

## 9 Vicious cycle of checkpoint molecules and drug-resistant bacterial infections

**Figure 3:**
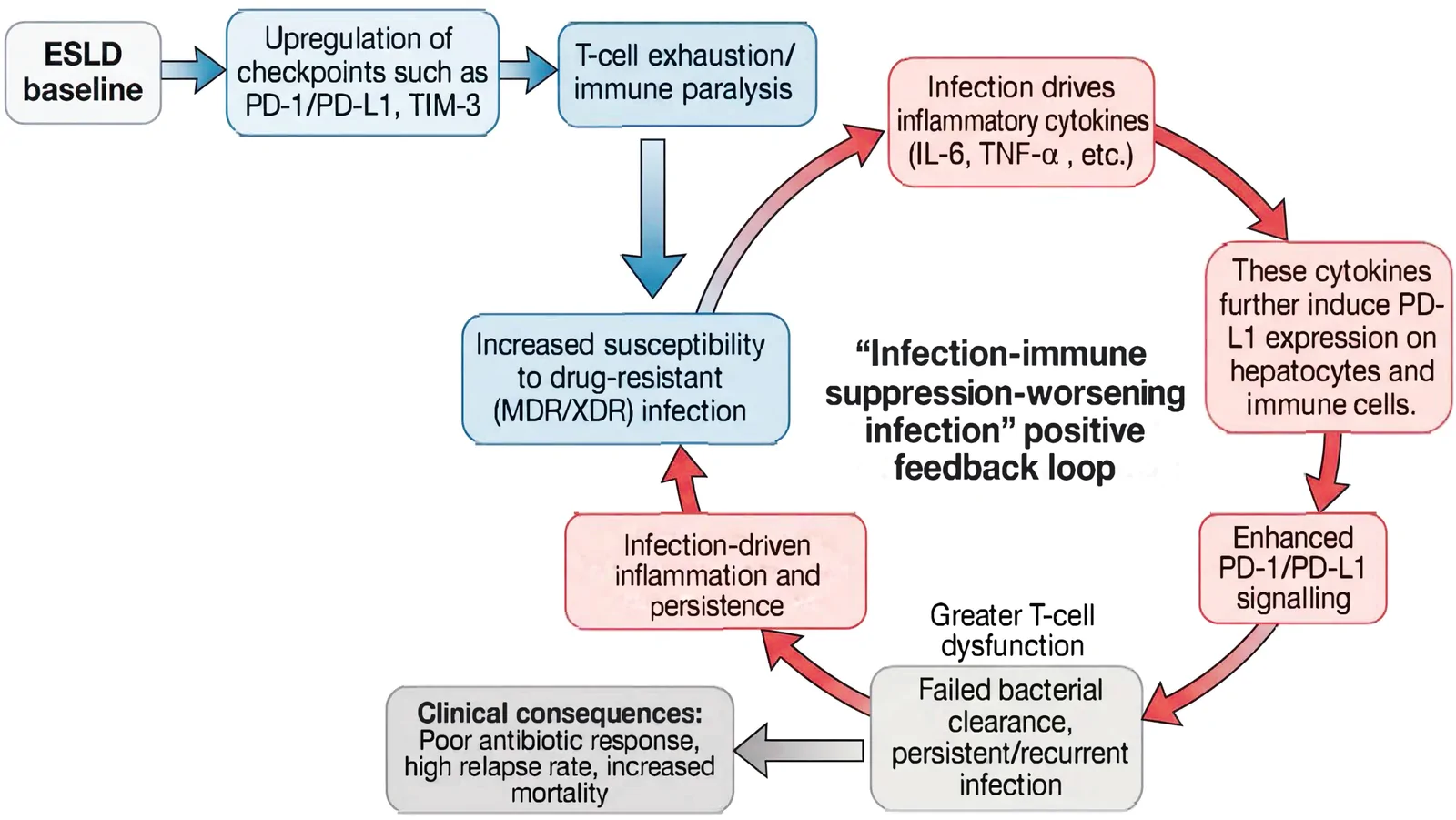
Vicious cycle of immune checkpoint molecules and drug−resistant bacterial infections in ESLD. The diagram illustrates a positive feedback loop through which immune checkpoint dysregulation and drug-resistant infection perpetuate each other in ESLD. At baseline, ESLD is characterized by upregulation of inhibitory checkpoint molecules such as PD-1, PD-L1, and TIM-3 on immune cells. This checkpoint overexpression drives T-cell exhaustion and immune paralysis, which in turn increases susceptibility to MDR and XDR infections. Once an infection establishes, it releases pro-inflammatory cytokines including IL-6 and TNF-α. These cytokines further induce PD-L1 expression on hepatocytes and other immune cells, amplifying checkpoint signaling. Enhanced PD-1/PD-L1 engagement leads to greater T-cell dysfunction, which impairs bacterial clearance and allows infection to become persistent or recurrent. This persistent infection sustains the inflammatory milieu, which continues to drive PD-L1 upregulation, thereby closing the loop. The resulting “infection−immune suppression−worsening infection” cycle has direct clinical consequences: poor response to antibiotic therapy, high rates of infection relapse, and increased mortality.

The pre-existing immunosuppressive environment in ESLD, characterized by upregulated immune checkpoints, creates a vicious cycle when challenged by drug-resistant bacterial infections. The infection itself acts as a potent secondary stressor, further exacerbating immune dysfunction. While direct evidence in ESLD patients with bacterial infections is limited, mechanistic insights from other chronic inflammatory and infectious models are informative. In multidrug−resistant tuberculosis (MDR−TB), systemic and Mycobacterium tuberculosis−induced Th22 responses are low and inversely correlate with a high proportion of senescent PD−1+ T cells; blockade of the PD−1/PD−L1 pathway *in vitro* enhances IL−22+ cell expansion, indicating that checkpoints actively suppress protective immunity during chronic MDR infection (79). This paradigm suggests that in ESLD, a drug-resistant bacterial infection could similarly reinforce checkpoint-mediated suppression. The infection-driven inflammatory cytokines may further induce PD-L1 expression on hepatocytes and immune cells, leading to more profound T cell exhaustion. This establishes a detrimental feedback loop: baseline immune suppression in ESLD predisposes to colonization by MDR organisms; the resulting infection deepens immunosuppression by amplifying checkpoint molecules, creating a cycle of “infection−immune suppression−worsening infection.” Beyond checkpoint dysregulation, the inflammatory burst from infection can also inflict direct hepatic damage via additional molecular pathways. For instance, the long non-coding RNA TTN-AS1 is upregulated in sepsis and promotes acute liver injury, suggesting such molecules could serve as potential monitoring biomarkers and therapeutic targets to interrupt the vicious cycle of infection and hepatic decompensation (80). This exhausted and injured state has direct clinical ramifications, partially explaining the observed poor and delayed response to antibiotic therapy and the high rate of infection recurrence in ESLD patients. Effective clearance of bacteria requires a synergistic effort between antimicrobial drugs and a competent host immune system for phagocytosis and pathogen elimination. When T cells are functionally exhausted due to chronic PD−1/PD−L1 signaling, this essential clearance component fails. Even if antibiotics reduce the bacterial burden, the impaired immune system cannot fully eradicate MDR pathogens or prevent relapse. This disconnect between pharmacological therapy and immune competence underscores why infections in ESLD are particularly recalcitrant. The situation is analogous to observations in cancer immunotherapy: in HCC patients treated with atezolizumab/bevacizumab, early mortality is associated with impaired liver function and systemic immune alterations, including reduced lymphocyte frequencies (81). This highlights how advanced liver disease and its associated immune dysfunction critically undermine the capacity to mount a beneficial immune response, whether against tumors or, by extension, against drug−resistant infections (**Figure 3**).

## 10 Immunomodulation and reconstruction strategies

The management of ESLD complicated by MDR infections necessitates a paradigm shift beyond conventional antimicrobial therapy, focusing on immune modulation and reconstitution strategies to restore host defense mechanisms (**Table 1**). A primary area of investigation involves the cautious targeting of immune checkpoints. The rationale is to reverse the T-cell exhaustion commonly observed in chronic liver disease and severe infections, thereby potentially restoring antimicrobial immunity. Agents like PD-1/PD-L1 inhibitors are under early exploration for this purpose (82). However, their application in ESLD patients with active infections requires extreme prudence due to the dual risk of precipitating immune-related adverse events, such as severe hepatitis flare-ups, and the theoretical concern of exacerbating infection through excessive immune activation. This delicate balance underscores the need for stringent patient selection and risk assessment in clinical trials. Another promising avenue is cytokine and cellular therapy. The administration of granulocyte-macrophage colony-stimulating factor (GM-CSF) aims to bolster the function and number of myeloid cells, which are often dysfunctional in ESLD, to enhance phagocytosis and bacterial clearance. In a randomized trial of cirrhotic patients with refractory spontaneous bacterial peritonitis, adding GM−CSF to meropenem improved early response and 7−day survival (83). Concurrently, adoptive cell transfer therapies, such as infusing cytokine-induced killer (CIK) cells, are being studied (84). These ex vivo activated autologous or allogeneic immune cells are designed to directly target pathogens and potentially modulate the immunosuppressive microenvironment, though clinical data in ESLD remain limited. Furthermore, the use of immunostimulants like thymosin α1 is considered for its potential to improve T-cell function and proliferation. In an open−label randomized controlled trial of 73 patients with HBV−related ACLF, Tα1 treatment significantly increased 90−day transplant−free survival (85). Mechanistically, Tα1 rebalanced the immune response by modulating T−cell differentiation and cytokine productionmitigating excessive late−stage inflammation while preserving early immune activation, thereby breaking the cycle of hyperinflammation and immunosuppression that drives ACLF progression. While preclinical and some clinical data in sepsis and liver failure suggest benefits, the precise efficacy and optimal application of thymosin α1 specifically in the context of ESLD with MDR bacterial infections lack robust, high-quality clinical evidence. Large-scale, randomized controlled trials are essential to determine its role, timing, and dosing regimen within a comprehensive antimicrobial and supportive care framework. These immunomodulatory approaches represent a move towards restoring a functional immune response, but their integration into clinical practice hinges on demonstrating a clear safety profile and survival benefit in this critically ill patient population (86).

**Table 1:** Key mechanisms of immune dysfunction in end-stage liver disease (ESLD) and potential intervention strategies.

Immune domain	Major pathological changes	Contribution to MDR infections	Potential intervention strategies
Innate immunity	KC depletion (PANoptosis), PD-1 upregulation, complement deficiency, monocyte dysfunction	Failure of the intravascular “firewall,” allowing MDR pathogens to evade immune clearance	PD-1/PD-L1 blockade; GM-CSF to enhance myeloid function
Adaptive immunity	T-cell exhaustion (PD-1↑, TIM-3↑), Treg/Th17 imbalance, low-affinity B cell antibodies	Chronic colonization and immune evasion by MDR pathogens	Immune checkpoint inhibitors; adoptive CIK cell therapy; thymosin α1
Gut–liver axis disruption	Gut barrier breakdown, dysbiosis (SCFA producers↓), endotoxin translocation	MDR colonization → translocation → SBP/bacteraemia	Probiotics/prebiotics; FMT; selective digestive decontamination
Immunometabolic reprogramming	Hyperammonaemia, lactate accumulation, M2 macrophage polarization, NK cell dysfunction	“Energy starvation” of immune cells, enabling MDR pathogen persistence	Ammonia-lowering therapy (rifaximin/L-ornithine L-aspartate); metabolic intermediate supplementation
Immune checkpoint dysregulation	PD-1/PD-L1, TIM-3, CTLA-4 upregulation; MDSC expansion	T-cell exhaustion → failed bacterial clearance → infection relapse	Immune checkpoint inhibitors (with careful risk assessment)

KC, Kupffer cell; PD-1, programmed cell death protein 1; PD-L1, programmed death-ligand 1; TIM-3, T-cell immunoglobulin and mucin domain-containing protein 3; CTLA-4, cytotoxic T-lymphocyte-associated protein 4; GM-CSF, granulocyte-macrophage colony-stimulating factor; CIK, cytokine-induced killer; SCFA, short-chain fatty acid; FMT, fecal microbiota transplantation; SBP, spontaneous bacterial peritonitis; MDSC, myeloid-derived suppressor cell.

↑ denotes an increase or upregulation (e.g., PD‑1↑, TIM‑3↑ indicates increased expression of these checkpoint molecules); ↓ denotes a decrease or depletion (e.g., SCFA producers↓ indicates reduced abundance of short‑chain fatty acid‑producing taxa); and → denotes a progression or causal relationship (e.g., MDR colonization → translocation → SBP/bacteraemia indicates the sequential path from colonization to infection).

## 11 Microecology and metabolic intervention

Correcting the profound dysbiosis and associated metabolic disturbances is a cornerstone of emerging strategies to manage ESLD and its infectious complications. The gut-liver axis is severely compromised in ESLD, leading to bacterial translocation, endotoxemia, and a pro-inflammatory state that fuels immune dysfunction and increases susceptibility to MDR organisms. Precision modulation of the microbiome is therefore a critical intervention target. Strategies range from selective digestive decontamination (SDD) and the use of specific probiotics and prebiotics to more aggressive approaches like fecal microbiota transplantation (FMT). The goal is to restore a healthier microbial community, competitively exclude pathogenic and MDR bacteria from colonization, strengthen the intestinal barrier, and thereby reduce bacterial translocation. In cirrhotic patients, adjuvant probiotic therapy has shown potential to restore microbial balance and improve clinical outcomes, supporting the rationale for microbiome-directed interventions (36). The ChiFT trial, a Danish multicentre, randomized, placebo-controlled trial, is currently investigating whether FMT can improve the disease course in patients with acute decompensation of liver cirrhosis, which will provide high-quality evidence on the clinical utility of this approach (87). This indirect restoration of gut-liver axis homeostasis can ameliorate systemic inflammation and potentially improve the host’s immune competence. Notably, beyond taxa-level changes, specific microbial metabolites have been directly linked to host metabolic health. For example, decreased levels of Flavonifractor plautii and its metabolite phytosphingosine have been associated with metabolic disorders, highlighting the importance of restoring both microbial composition and metabolic function (37). Evidence highlights that patients with ESLD, including those awaiting or having undergone liver transplantation, exhibit significant gut dysbiosis. This dysbiosis is further exacerbated by repeated antibiotic courses and is closely linked to infectious outcomes (42, 88). Parallel to microbiome-focused interventions, metabolic regulation therapy offers another lever for immune modulation. Controlling hyperammonemia with agents like rifaximin or L-ornithine L-aspartate is standard, but these treatments may confer additional immunologic benefits by reducing ammonia’s direct suppressive effects on immune cell function. More novel is the exploration of correcting the intrinsic metabolic defects in immune cells. Supplementing specific metabolic intermediates (e.g., L-carnitine for fatty acid oxidation) or employing pharmacological agents to reprogram immune cell metabolism from an immunosuppressive to an effector state is an emerging research frontier. This approach aims to directly fuel immune cells, enhancing their antimicrobial capacity. The interplay between microbial metabolites and host immunity extends beyond SCFAs; metabolites such as phytosphingosine have been shown to influence metabolic and inflammatory pathways, and their depletion may contribute to immune dysfunction in ESLD (37). This interplay is particularly relevant post-transplantation, where metabolites can significantly influence immune cell function and inflammatory responses (86). Thus, combined microecological and metabolic interventions represent a holistic strategy to reshape the host’s internal environment, making it less permissive for MDR infection establishment and progression.

## 12 Integration of new treatment models

The future of managing ESLD with MDR infections lies in the integrated application of novel therapeutic modalities, moving from a purely antimicrobial approach to a personalized “anti-pathogen plus immune adjuvant” combination strategy. This paradigm recognizes that eradicating the pathogen is insufficient if the host’s immune system remains paralyzed. Therefore, synergistic combinations of antibiotics with immunomodulatory agents tailored to the patient’s specific immune dysfunction profile are under development. For example, a Cochrane review of 12 randomized controlled trials (823 patients) evaluated stem cell therapy for decompensated cirrhosis, including bone marrow−derived mesenchymal cells, mononuclear cells, and umbilical cord stem cells. Mortality was 13.3% with stem cells versus 29% in controls (seven studies), and serious adverse events occurred in 20.2% versus 34.6% (eight studies). However, evidence certainty was very low due to methodological limitations, so stem cell therapy remains experimental pending further rigorous evaluation (89).

Crucially, the success of this integrated model hinges on the identification and validation of reliable biomarkers that can accurately reflect the degree and type of immune impairment in individual ESLD patients. Potential biomarkers include specific cytokine profiles (e.g., ratios of pro-inflammatory to anti-inflammatory cytokines), detailed immunophenotyping of circulating and tissue-resident immune cells (revealing exhaustion markers, functional subsets), and distinct metabolomic or microbiome signatures. In acutely decompensated cirrhosis, fecal cytokine profiling has been shown to reflect intestinal inflammation and correlate with systemic inflammatory markers and disease severity, supporting the utility of non−invasive biomarker approaches in this population (53). The composition of the gut or biliary microbiome itself can serve as a biomarker for infection risk and immune status, as dysbiosis is closely linked to vulnerability to MDR bacteria and post-transplant outcomes. These biomarkers would guide clinicians in selecting the most appropriate immunomodulatory intervention, whether it be checkpoint inhibition, cytokine therapy, or microbiome restoration, and determining the optimal timing for its initiation. Furthermore, understanding pharmacomicrobiomics, or how the microbiome metabolizes drugs, is crucial for personalizing immunosuppressive and antimicrobial regimens in transplant settings. The gut microbiome can modulate drug efficacy and toxicity through direct enzymatic conversion of immunosuppressive agents, and pharmacomicrobiomic profiling has been proposed as a tool to optimize individualized dosing strategies (90). Microorganisms can alter the efficacy of drugs like tacrolimus, impacting both immunosuppression and infection risk (90). This biomarker-guided, integrated approach promises a more precise and effective therapeutic framework. It envisions a treatment pathway where initial assessment includes deep immune and microbial profiling, followed by a dynamically adjusted regimen combining targeted antibiotics, immune-reconstituting agents, and microbiome/metabolic therapies, all monitored through longitudinal biomarker assessment to optimize outcomes and minimize complications in this high-risk population.

## 13 Discussion

The findings summarized in this review highlight a central observation: the heightened susceptibility of ESLD patients to drug-resistant bacterial infections arises from a synergistic interplay of multiple immune defects, including innate and adaptive immune dysfunction, gut-liver axis disruption, immunometabolic reprogramming, and immune checkpoint dysregulation. While previous work has largely addressed these mechanisms in isolation, this review distinguishes itself by weaving them into a unified framework that shows how they converge to drive MDR pathogen colonization, persistence, and treatment failure. We identify checkpoint upregulation, metabolic shifts, and gut-derived signals as key actionable nodes within this network. These nodes are not independent; checkpoint molecules such as PD-1 and PD-L1 lock T cells in exhaustion, metabolic disturbances like hyperammonemia and lactate accumulation cripple effector functions, and gut-derived endotoxins perpetuate systemic inflammation. Targeting any one of these nodes may disrupt the cycle, but simultaneous, personalized modulation could yield greater therapeutic benefit. By bridging mechanistic understanding with clinical translation, our framework provides a foundation for biomarker discovery and the design of adjunctive immunotherapies that complement antimicrobial treatment in this high-risk population.

A central observation is that immune dysfunction in ESLD is not merely quantitative but qualitatively reprogrammed, creating a paradoxical state of concurrent inflammation and paralysis. The depletion and functional impairment of KCs via PANoptosis and PD-1 upregulation (20–22) directly compromises the liver’s intravascular firewall, a concept showing gut microbiota-dependent programming of this niche (19). However, whether KC dysfunction precedes or follows bacterial translocation in ESLD remains unclear; longitudinal studies in well-defined cohorts are needed. Similarly, complement deficiency due to synthetic failure (34) likely exacerbates susceptibility to Gram-negative MDR strains, yet no studies have systematically quantified complement opsonization capacity against ESBL-producing Enterobacterales in ESLD patients.

The gut-liver axis emerges as a critical amplifier of immune failure. Persistent endotoxemia from bacterial translocation induces TLR4 tolerance in KCs (49), while dysbiosis reduces SCFA production (36), removing essential anti-inflammatory signals (51). This dual hit, continuous pro-inflammatory stimulation coupled with loss of regulatory metabolites, may explain the endotoxin tolerance phenotype described in cirrhosis (51). Importantly, the role of specific microbial metabolites like butyrate in directly modulating T-cell metabolism and checkpoint expression in ESLD remains underexplored. Recent work in transplantation (86) suggests that tacrolimus further disrupts microbiota composition and metabolite profiles (54), potentially worsening MDR colonization post-LT. This is a clinically actionable insight requiring prospective validation.

Immunometabolic reprogramming represents a newly appreciated driver of immune paralysis. Hyperammonemia inhibits T-cell glycolysis and glutaminolysis (58), while lactate accumulation skews macrophages toward an M2-like, immunosuppressive phenotype (71, 72) and impairs NK cell cytotoxicity (68). These metabolic defects are reminiscent of the tumor microenvironment (73, 74) and may explain why ESLD patients fail to clear MDR pathogens even when antibiotic susceptibility is preserved. However, direct evidence linking specific metabolite levels to defined immune functional assays in ESLD with active MDR infection is lacking. Such correlative studies are urgently needed to establish threshold effects and therapeutic windows.

Checkpoint molecule upregulation, particularly PD-1/PD-L1, further reinforces T-cell exhaustion (73, 74). The proposed vicious cycle of infection leading to inflammatory cytokines, then PD-L1 induction, then deeper exhaustion, then impaired clearance, and finally persistent infection is mechanistically plausible but currently inferred from models of MDR-TB (79) and HCC immunotherapy (81). Direct evidence in ESLD patients with bacteremia or spontaneous bacterial peritonitis caused by MDR organisms is sparse. Single-cell sequencing of paired blood and ascitic fluid immune cells before and during infection could resolve this gap. The identification of novel molecular players in sepsis-induced liver injury further expands the therapeutic landscape. For instance, the long non-coding RNA TTN-AS1 has been shown to promote acute liver injury in sepsis, and targeting such molecules may offer new avenues for monitoring disease progression and intervening at the intersection of infection and organ failure (80). Whether similar mechanisms operate in ESLD patients with MDR infections remains to be investigated.

The contribution of gut microbiota to ESLD progression also varies across different underlying etiologies. In patients with HBV-related end-stage liver disease, metagenomic and metabolomic analyses of liver tissues have revealed significant compositional and functional reprogramming of the intrahepatic microbiome, characterized by altered microbial diversity and functional pathways coupled with perturbations in liver metabolism, immune responses, and structural remodeling. These findings suggest that the intrahepatic microbiome may actively contribute to disease progression in HBV-related ESLD through local metabolic and immune modulation, rather than merely reflecting passive translocation from the gut (91). Beyond its role in MDR colonization, the gut microbiome has also emerged as a potential biomarker for post-transplant management. Microbiome profiling could inform personalized adjustment of immunosuppressive therapy to balance rejection prevention against infection susceptibility, representing an evolving frontier in transplant medicine (92). Clinically, the current therapeutic paradigm remains heavily antibiotic−centric, yet the host’s inability to mount effective immunity predicts poor outcomes regardless of antimicrobial susceptibility (50, 93). Several adjunctive strategies have been explored to address this immune deficit, though each faces substantial hurdles. PD−1/PD−L1 blockade can restore immune function in preclinical models, but real−world pharmacovigilance data indicate a risk of immune−mediated liver injury and hepatic failure, limiting its use in ESLD patients outside clinical trials (81). GM−CSF has been shown to reduce myeloid−derived suppressor cells and improve T−cell function in decompensated cirrhosis with sepsis (86). Clinical evidence further supports its potential: GM−CSF improved early response and short−term survival in difficult−to−treat spontaneous bacterial peritonitis (83), while granulocyte colony stimulating factor reduced sepsis risk and improved survival in decompensated cirrhosis, though benefits were predominantly observed in Asian studies (94). Thymosin α1 has shown efficacy in chronic liver failure with sepsis and in hepatitis B cirrhosis with spontaneous bacterial peritonitis, but no study has specifically examined its effect in ESLD patients with MDR infections (88, 95). Fecal microbiota transplantation can lower the antibiotic resistance gene burden in cirrhosis, though engraftment is variable and the risk of bacteremia remains a concern in patients with a compromised gut barrier (42, 96). Metabolic correction may offer immunologic benefits, but no trial has used immune restoration as a primary endpoint. To guide patient selection for these interventions, several biomarkers warrant prospective evaluation: soluble PD−L1 levels correlate with infection risk and mortality in chronic liver disease; fecal butyrate concentration predicts 30−day transplant−free survival in decompensated cirrhosis; and the ammonia−to−lactate ratio has been proposed as an indicator of immunometabolic dysfunction. Looking ahead, a clinical algorithm could begin with baseline immune profiling that includes T−cell exhaustion markers, monocyte HLA−DR expression, and gut microbial composition. This information would guide the selection of targeted immunotherapy to be used alongside appropriate antimicrobials, with longitudinal monitoring to adjust treatment as the patient’s immune status evolves.

Several limitations should be kept in mind when interpreting the evidence summarized here. Most studies are cross−sectional or retrospective. They can generate hypotheses and point to associations, but they cannot tell us whether a given immune defect comes before or after the infection. We need longitudinal studies that track immune function, microbiota, and clinical outcomes over time to sort out cause and effect. Definitions of MDR infections also vary widely. Some studies rely on antimicrobial susceptibility panels, others use clinical criteria, and still others focus on specific infection sites. This makes it hard to compare results across studies and may explain why reported MDR prevalence ranges so broadly. Another issue is the lack of validated immune phenotyping panels for ESLD. We have assays for T−cell function, HLA−DR expression, and cytokine profiling in other settings, but we do not know whether they perform the same way in ESLD patients, nor what thresholds define clinically meaningful immune paralysis. Finally, most data come from Europe, North America, and Asia. Low− and middle−income countries, where resistance rates are highest, are underrepresented. This geographic imbalance may hide region−specific mechanisms and make our findings less generalizable.

To move forward, we need prospective multicenter cohorts that collect clinical, immune, metabolomic, and microbiome data over time. Such studies would allow us to establish temporal sequences, capture the dynamics of immune dysfunction as liver disease progresses, and include the full spectrum of ESLD—especially ACLF patients. Biomarker validation is another priority. Soluble PD−L1, fecal butyrate, and the ammonia−to−lactate ratio are promising candidates, but none has been prospectively tested in ESLD for predicting MDR infection risk or guiding therapy. We need to establish whether they work in this population and whether using them to guide decisions actually improves outcomes. Clinical trials of immune−modulating therapies are overdue. Checkpoint inhibitors, GM−CSF, G−CSF, thymosin α1, and FMT have shown potential in preclinical or early studies, but we know very little about their safety and efficacy in ESLD patients with active MDR infections. Given the fragility of this population, these trials will need tight safety monitoring for hepatitis flares and infection exacerbation. Finally, mechanistic work using humanized mouse models or organoids should be pursued to examine how checkpoint pathways, metabolism, and infection interact. These systems can help us understand causal mechanisms that are difficult to study in patients and test potential targets before moving to clinical trials. If we can integrate immunomodulation into routine care, we will need to move from a pathogen−centered approach to a host−restoring one, guided by dynamic immune monitoring and personalized regimens. That shift will require basic scientists, clinicians, and health systems to work together in translating mechanistic insights into practical strategies that improve outcomes for this high−risk group.

## 14 Conclusion

The susceptibility of patients with ESLD to multidrug-resistant organisms is not simply a consequence of liver failure. It reflects a complex web of immune dysfunction that affects nearly every component of host defense. Innate immunity falters. Kupffer cells are depleted, complement proteins run low, and circulating monocytes lose their capacity to respond to microbial challenge. Adaptive immunity is similarly compromised. T cells become exhausted under persistent antigenic stimulation, regulatory T cells expand at the expense of protective effector subsets, and B cells produce antibodies that bind but fail to opsonize effectively. Meanwhile, the intestinal barrier breaks down, allowing bacterial products to flood the portal circulation and sustain low-grade systemic inflammation. Metabolic disturbances such as hyperammonemia and lactate accumulation further suppress immune cell function. Checkpoint molecules like PD-1 and PD-L1 become overexpressed, locking T cells in a state of dysfunction and creating a self-perpetuating loop in which infection deepens immunosuppression, and immunosuppression invites further infection. These mechanisms are not independent. They reinforce one another, collectively shaping an environment that not only allows resistant pathogens to colonize and evade clearance but also makes these infections exceptionally difficult to treat.

From a clinical standpoint, this understanding challenges the current therapeutic paradigm. For decades, management of infections in ESLD has been centered on antimicrobial agents, choosing the right drug, adjusting for altered pharmacokinetics, and hoping that the host immune system will clear the rest. But the evidence reviewed here suggests that this approach is fundamentally incomplete. When the host is unable to mount an effective immune response, even the most potent antibiotics may fail to eradicate the pathogen or prevent recurrence. The mechanistic framework outlined in this review points toward a fundamental shift in clinical strategy: moving from “treat the pathogen” to “restore the host.” This does not mean abandoning antimicrobial therapy, but rather recognizing that it is only half of the equation. Future investigative efforts must prioritize several interconnected goals: elucidation of the network interactions between different immune mechanisms, validation of biomarkers that can stratify infection risk and predict therapeutic response, and rigorous evaluation of immunomodulatory adjunctive therapies in well-designed clinical trials.

The long-term goal is to translate mechanistic insight into immunomodulatory strategies that complement and enhance antimicrobial therapy. This will not be easy. ESLD patients are fragile, and immune modulation carries real risks, including hepatitis flares and infection exacerbation. But the potential benefit is substantial. Achieving this goal will require close collaboration among basic immunologists, clinical hepatologists, infectious disease specialists, and pharmacologists. It will also require investment in translational research that bridges laboratory discoveries and bedside applications. Only through such a comprehensive, mechanism-based, and personalized approach can we break the cycle of infection and immune failure and improve the prognosis for this high-risk population.
